# Pruritic scaly rash of the inframammary folds

**DOI:** 10.1016/j.jdcr.2024.11.001

**Published:** 2024-11-14

**Authors:** Daphne G. Eckembrecher, Jacob Dudzinski, Vesna Petronic-Rosic

**Affiliations:** a Division of Dermatology, University of Miami Miller School of Medicine, Holy Cross Health, Fort Lauderdale, Florida; b Division of Dermatology, John H. Stroger Hospital of Cook County, Chicago, Illinois

**Keywords:** inframammary fold, pruritus, rash

A 65-year-old female presented with a rash in the bilateral inframammary folds for 3 months. She complained of itching and burning in the affected area. Her past medical history was notable for type 2 diabetes mellitus, hypertension, and transient ischemic attack. On physical examination, the inframammary folds had well-demarcated hyperkeratotic brown scaly papules coalescing into plaques (Fig 1). A skin biopsy was performed (Fig 2. H&E, 20x).

**Fig 1:**
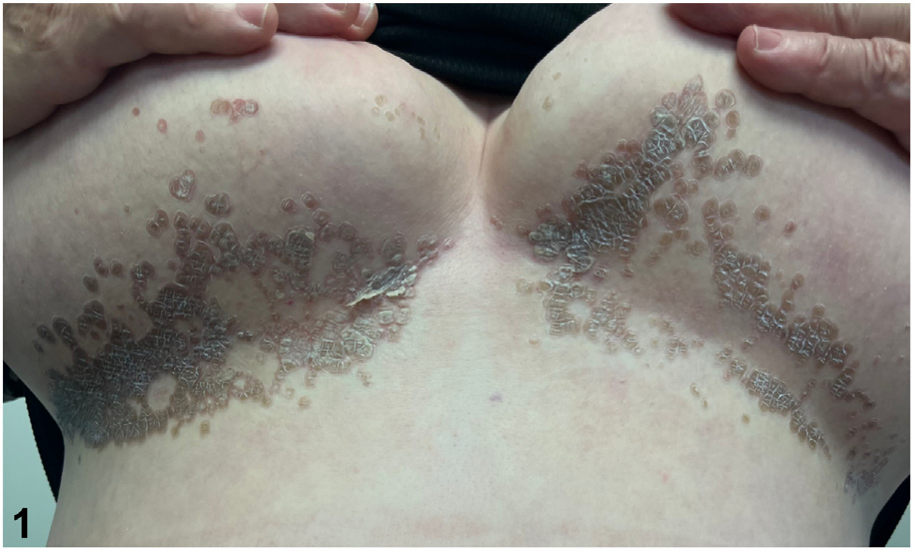

**Fig 2:**
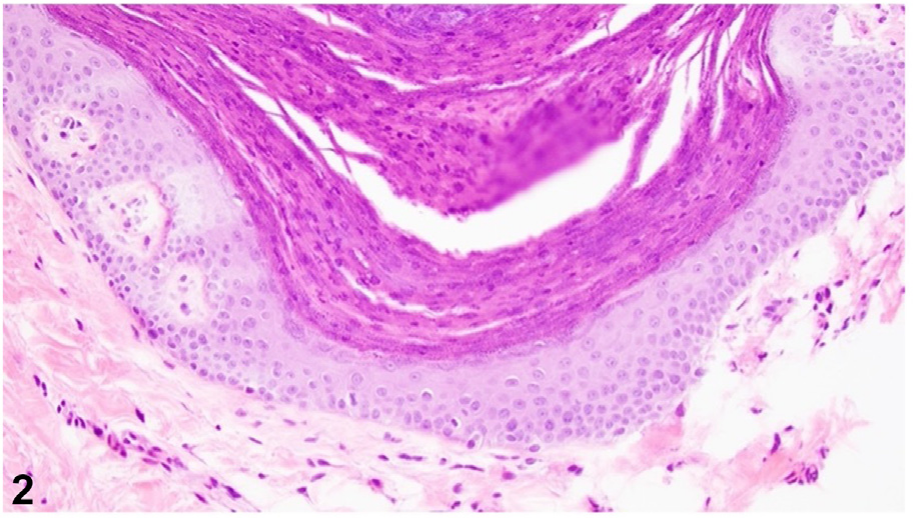

**Question 1: Which of the following is the most likely diagnosis?**

A. Hailey-Hailey disease
B. Erythrasma
C. Granular parakeratosis
D. Inverse psoriasis
E. Darier disease

**Answers:**

A. Hailey-Hailey disease – Incorrect. Also known as benign familial pemphigus, it is a rare autosomal dominant disorder characterized by impaired keratinocyte adhesion, which results in intraepidermal acantholysis. The typical locations affected include the sides and back of the neck, axillary, inguinal, and perineal folds.^1^
B. Erythrasma – Incorrect. A bacterial skin infection caused by gram-positive *Corynebacterium minutissimum.* It presents as reddish-brown scaly macules and patches most commonly in the inguinal folds.^2^
C. Granular parakeratosis – Correct. This is a rare disease; the exact pathogenesis is unknown and the etiology is not well understood. It is characterized by reddish-brown hyperkeratotic papules and scaly erythematous patches. Granular parakeratosis occurs in the flexures and occluded sites, most commonly in the axillae.^3^
D. Inverse psoriasis – Incorrect. Inverse psoriasis is characterized by moist red plaques occurring in body folds, most commonly in the anogenital, axillary, and inframammary regions.^4^
E. Darier disease – Incorrect. Also known as follicular dyskeratosis, it is a rare autosomal dominant disorder characterized by reddish-brown confluent papules in seborrheic areas and partly macerated papillomatous plaques in intertriginous areas.^5^

**Question 2: What is the most common risk factor for developing this condition?**

A. Topical agents
B. Mechanical stress
C. Bacterial infection
D. Humidity
E. Secondary infection

**Answers:**

A. Topical agents – Correct. The most common culprit for the development of granular parakeratosis are topical agents containing zinc oxide, deodorants/antiperspirants, and benzalkonium chloride.^3^
B. Mechanical stress – Incorrect. Hailey-Hailey and Darier disease can be triggered by mechanical stress; however, it is not the most common risk factor for granular parakeratosis.^5^
C. Bacterial infection – Incorrect. Bacterial infection caused by *Corynebacterium minutissimum* is associated with the development of erythrasma.^2^
D. Humidity – Incorrect. Humidity is a trigger for the development of inverse psoriasis.^4^
E. Secondary infection – Incorrect. Secondary infections are a risk factor for the development of Hailey-Hailey disease.^1^

**Question 3: Which of the following are histologic features of this condition?**

A. Epidermal hyperplasia with elongation of the rete ridges
B. Keratinocyte acantholysis appearing as a “dilapidated brick wall”
C. Hyperkeratosis and parakeratosis with retained keratohyalin granules within the stratum corneum, psoriasiform or papillomatous epidermal hyperplasia, and a lymphocytic infiltrate
D. Dyskeratotic cells in the stratum spinosum which appear as “corps ronds”
E. Spongiotic dermatitis with parakeratosis at the shoulders of follicular ostia

**Answers:**

A. Epidermal hyperplasia with elongation of the rete ridges – Incorrect. Epidermal hyperplasia with elongation of the rete ridges is a histologic presentation of inverse psoriasis.^4^
B. Keratinocyte acantholysis appearing as a “dilapidated brick wall” – Incorrect. Keratinocyte acantholysis that appears as a “dilapidated brick wall” is a histologic hallmark of Hailey-Hailey disease.^1^
C. Hyperkeratosis and parakeratosis with retained keratohyalin granules within the stratum corneum, psoriasiform or papillomatous epidermal hyperplasia, and a lymphocytic infiltrate – Correct. Granular parakeratosis presents with hyperkeratosis and parakeratosis with retained keratohyalin granules within the stratum corneum, psoriasiform or papillomatous epidermal hyperplasia, and a lymphocyte predominant interstitial or perivascular infiltrate in the superficial dermis.^3^
D. Dyskeratotic cells in the stratum spinosum which appear as “corps ronds” – Incorrect. Dyskeratotic cells in the stratum spinosum which appear as “corps ronds” are a feature of Darier disease.^5^
E. Spongiotic dermatitis with parakeratosis at the shoulders of follicular ostia – Incorrect. Spongiotic dermatitis with parakeratosis at the shoulders of follicular ostia is a common presentation of seborrheic dermatitis.^5^

## Conflicts of interest

None disclosed.
